# Humoral and Cellular CMV Responses in Healthy Donors; Identification of a Frequent Population of CMV-Specific, CD4+ T Cells in Seronegative Donors

**DOI:** 10.1371/journal.pone.0031420

**Published:** 2012-02-07

**Authors:** Nina Loeth, Kristian Assing, Hans O. Madsen, Lars Vindeløv, Soren Buus, Anette Stryhn

**Affiliations:** 1 Laboratory of Experimental Immunology, Faculty of Health Sciences, University of Copenhagen, Copenhagen, Denmark; 2 Blood Bank, Rigshospitalet, Copenhagen, Denmark; 3 The Tissue Typing Laboratory, Department of Clinical Immunology, Rigshospitalet, Copenhagen University Hospital, Copenhagen, Denmark; 4 The Allogeneic Hematopoietic Cell Transplantation Laboratory, Department of Hematology, Rigshospitalet, Copenhagen University Hospital, Copenhagen, Denmark; University of Palermo, Italy

## Abstract

CMV status is an important risk factor in immune compromised patients. In hematopoeitic cell transplantations (HCT), both donor and recipient are tested routinely for CMV status by serological assays; however, one might argue that it might also be of relevance to examine CMV status by cellular (i.e., T lymphocyte) assays. Here, we have analyzed the CMV status of 100 healthy blood bank donors using both serology and cellular assays. About half (56%) were found to be CMV seropositive, and they all mounted strong CD8+ and/or moderate CD4+ T cell responses *ex vivo* against the immunodominant CMV protein, pp65. Of the 44 seronegative donors, only five (11%) mounted *ex vivo* T cell responses; surprisingly, 33 (75%) mounted strong CD4+ T cell responses after a brief in vitro peptide stimulation culture. This may have significant implications for the analysis and selection of HCT donors.

## Introduction

Human cytomegalovirus (CMV) is a ubiquitous β-herpes virus infecting 50–80% of the adult population [1], [2]. It rarely causes disease in immunocompetent individuals; rather, CMV establishes a life-long asymptomatic latent infection with intermittent sub-clinical reactivations, which are controlled by the immune system. In immunocompromised patients, however, reactivation of CMV can cause considerable morbidity and mortality especially after solid organ transplantation and allogeneic hematopoietic cell transplantation (HCT) [3], [4], [5], [6]. The risk and outcome of CMV reactivation is a particularly complicated issue in HCT due to the gross disturbance of the otherwise finely tuned balance between the viral burden (contributed by latent infections of either the recipient and/or the donor graft) and the immune system (suppressed and destined to be replaced by the donor immune system, which may or may not be CMV experienced). Reestablishing appropriate immune control of latent CMV infection depends upon the CMV statuses of the donor and the recipient [7] and strongly affects the outcome of the HCT [4], [8], [9].

Prior to implementation of effective anti-CMV drugs in the early 1990s, CMV disease (often presenting itself as CMV pneumonitis) used to be the leading infectious cause of death among CMV-seropositive recipients of HCT [4]. The implementation of preventive strategies encompassing prophylaxis and preemptive therapy [10] has reduced CMV disease during the first 3 months after HCT from 20–30% to less than 5% [10]. Despite of these accomplishments, establishing the CMV statuses of the HCT recipient and of the donor are still of considerable prognostic value for CMV reactivation and the outcome of HCT.

The CMV statuses of donor and recipient prior to HCT are routinely determined by serological testing for CMV-specific IgG and/or IgM antibodies [11]. However, CMV-specific T cells may be more important for immune protection against CMV reactivation and for long-term control of the virus [12], [13], [14], [15]. Thus, CMV reactivation occurs particularly frequently in seropositive HCT-recipients of T cell depleted grafts which often become refractory to antiviral therapy [4], [16], [17], and adoptive transfer of CMV-specific CD4+ and/or CD8+ positive T cells affords protection against CMV [18], [19], [20], [21]. Thus, establishing whether the donor is capable of raising a cellular response against CMV might be of considerable prognostic value.

Here, we have analyzed the CMV status of 100 healthy blood donors using a standard ELISA-driven serology test and in parallel a cellular test measuring intracellular cytokine secretion (ICS) in CMV-specific T cells.

## Results

### Establishing CMV status by serology

A commercial ELISA-based kit was used to determine total anti-CMV IgG and IgM antibodies in donor plasma of 100 anonymous healthy blood donors, aged 19 to 75. Of these 100 donors, 44 were CMV seronegative and 56 were CMV seropositive. There was a slightly lower median age distribution in the seronegative group (33.5 years) than in the seropositive group (40.5 years) (not significant, P = 0.15).

### Cellular CMV reactivity in seropositive or seronegative individuals

In general, antibodies recognize antigen structures. In contrast, T cells always recognize short peptide fragments derived from protein antigens and presented in the context of the highly polymorphic MHC molecules on the surface of antigen presenting cells. The blood donors were tested for the presence of CMV-specific T cell responses. Mixtures of overlapping peptides, e.g. 15 amino acid long peptides overlapping by 11 amino acids, may conveniently represent protein antigens. This peptide size and overlap optimize the chances of simultaneously generating both the longer (about 13 amino acid) CD4 T cell targets and the shorter (about 9 amino acid) CD8 T cell targets during the cell culture [22], [23]. As target protein we selected the CMV lower matrix phosphoprotein 65, pp65. It is one of the most highly expressed CMV proteins, and it is also one of the most dominant CD4 and CD8 T cell antigen derived from CMV [14].

**Figure 1:**
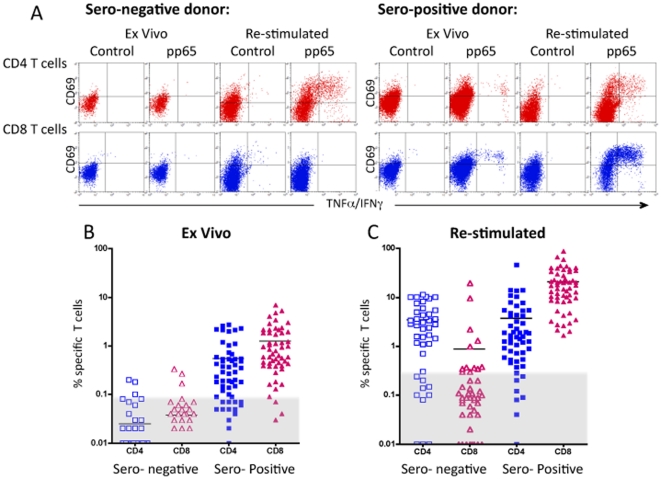
Detection of pp65 specific CD4+ and CD8+ T cell responses. PBMC from 100 healthy donors, 44 CMV seronegative and 56 seropositive, were analyzed either *ex vivo* or after *in vitro* stimulation with the pp65 peptide mixture for 7 days. The cells were examined for pp65 specific responses using the ICS assay and stained for CD4, CD8, CD69 and intracellular TNFα and IFNγ collectively. Background activation obtained in the absence of peptide was subtracted from the results obtained with the peptide mixture. In A) is shown examples of the ICS analysis of CD4 and CD8 T cell responses measures *ex vivo* and after stimulation of both a seropositive and a seronegative donor. In B) the *ex vivo* measured frequency of pp65 specific CD4 and CD8 T cells is shown for each seronegative and seropositive donor, respectively. In C) the frequency of pp65 specific CD4+ and CD8+ T cells measured after *in vitro* stimulation is shown for each seronegative and seropositive donor, respectively. After *in vitro* stimulation, the background activation was slightly higher than observed *ex vivo*; positive responses were defined as frequencies >0.09% for the *ex vivo* analysis, and as frequencies >0.3% for the *in vitro* stimulation analysis. Background frequencies have been subtracted the specific frequencies and responses above 0.09% for the ex vivo analysis and 0.3% for the in vitro analysis were considered positive. Shown as the responses above the grey areas.

T cells stimulated with overlapping pp65 peptide mixtures were analyzed by ICS to determine the phenotype (CD4 versus CD8), activation status (CD69) and functionality (IFNγ and TNFα secretion) of the responding T cells. Due to the intermittent reactivation of the latent CMV infection, CMV-specific T cells can be found in sufficiently high frequencies (at least for some epitopes) to enable direct *ex vivo* detection. However, to assure that any low frequency T cell responses were not missed, pp65 specific T cell responses were analyzed both *ex vivo* and after a brief *in vitro* stimulation culture. Examples of the ICS analysis *ex vivo* and after *in vitro* stimulation of a seronegative and of a seropositive donor are shown in Figure 1A. The seropositive donor of the example exhibited *ex vivo* CMV-specific CD4 and CD8 T cell responses, whereas the seronegative donor of the example only exhibited CD4 T cell responses after *in vitro* stimulation. The frequency data of all 100 donors is shown in Figure 1B (ex vivo responses) and Figure 1C (after in vitro stimulation). It is readily apparent that the vast majority of seropositive donors gave strong *ex vivo* CD4 and/or CD8 T cell responses, and this was even more pronounced after *in vitro* stimulation. In contrast, the vast majority of the seronegative donors gave no *ex vivo* CD4 and/or CD8 T cell responses. Surprisingly, 33 (75%) of the 44 seronegative donors gave a strong pp65 specific CD4 T cell response after in vitro stimulation.

A more detailed analysis of the *ex vivo* T cell responses (Table 1) showed that 56 (100%) of the 56 CMV seropositive donors exhibited *ex vivo* cellular responses against CMV pp65: 34 (61%) of the 56 seropositive donors gave both CD4 and CD8 T cell responses, 6 (11%) exhibited a CD4 T cell response only, and 16 (28%) exhibited a CD8 T cell response only. In contrast, only 5 (11%) of the 44 seronegative donors exhibited *ex vivo* cellular responses against CMV pp65: one donor gave both CD4 and CD8 T cell responses, two gave a CD4 T cell response only, and two gave a CD8 T cell response only. In 39 (89%) of the 44 seronegative donors, no ex vivo pp65-specific T cell responses could be detected.

**Table 1:** Ex vivo responses.

	Seronegative individuals		Seropositive individuals	
Ex vivo responses	#	%	#	%
No T cell responses	39	89	0	0
CD4 T cell responses only	2	4.5	6	11
CD8 T cell responses only	2	4.5	16	28
Both CD4 and CD8 T cell responses	1	2	34	61
Total	44	100	56	100

This picture changed considerably when the cells were briefly *in vitro* stimulated with a mixture of overlapping CMV pp65 peptides (Table 2). As before, all seropositive donors gave pp65 specific T cell response, albeit even stronger responses than *ex vivo*. Thus, all seropositive donors now gave detectable CD8 T cell responses, and as much as 47 (84%) of the 56 seropositive donors gave both CD4 and CD8 T cell responses. The most dramatic change, however, was observed for the seronegative donors: whereas only 5 (11%) of the 44 seronegative donors had exhibited pp65-specific T cell responses *ex vivo*, 34 (77%) of the same 44 seronegative donors exhibited pp65-specific T cell responses after pp65 peptide stimulation. This peptide stimulation T cell response was in particular driven by CD4 T cells: 33 (75%) of the 44 seronegative donors exhibited strong CD4 T cell response after peptide stimulation, 10 of these had also a CD8 T cell response, and only one of the 44 seronegative donors exhibited solely a CD8 T cell response after pp65 peptide stimulation.

**Table 2:** In vitro stimulated responses.

	Seronegative individuals		Seropositive individuals	
In vitro stimulated responses	#	%	#	%
No T cell responses	10	23	0	0
CD4 T cell responses only	23	52	0	0
CD8 T cell responses only	1	2	9	16
Both CD4 and CD8 T cell responses	10	23	47	84
Total	44	100	56	100

Thus, pp65-specific CD4 T cell responses dominated in the stimulated seronegative donor PBMC's. This was in clear contrast to the seropositive donors where CD8 T cell responses dominated, both in frequency of donors responding and in terms of frequencies of specific T cells within the individual donor. This picture of an apparent dichotomy of CD4 and CD8 T cell responses in seronegative and seropositive individuals, respectively, was reinforced when considering the strength of the responses.

The average frequency of specific T cells in the donors giving a detectable CD8 T cell response after *in vitro* stimulation was much higher in the seropositive than in the seronegative group (20,3% (range 0.6–87%) vs. 1.5% (range 0.3–9.8%), respectively, Figure 1B). In contrast, the CD4 responses in both groups were comparable after a *in vitro* stimulation culture (4,4% (range 0.4–46%) vs. 4.5% (range 0.3–11.4%), Figure 1C). Thus, seropositive individuals tended to give CD8 T cell responses, whereas seronegative individuals tended to give a CD4 T cell responses both in terms of frequency of donors responding and in terms of frequencies of specific T cells within the individual donors.

## Discussion

We have examined CMV serology and CMV pp65-specific T cell responses in 100 healthy blood bank donors. An established ELISA-driven assay was used to determine total anti-CMV IgG and IgM antibodies, and thereby the serological CMV status. This showed that about half (56%) of these donors were seropositive. Others have made similar findings [2], although variations can be considerable depending on several factors such as age, sex, ethnicity and socio-economic factors [24]. These donors were then examined for CD4 and CD8 T cell responses specific for a peptide mixture representing the immunodominant CMV protein, pp65, both measured ex vivo and after a brief in vitro stimulation culture. A very strong positive correlation was found between serology and ex vivo T cell responses defined as either a CD4 or a CD8 T cell response. By this token, every seropositive donor was identified by the cellular analysis, whereas only 5 (11%) of the seronegatives were identified (these could be categorized as false positives). Sester et al. observed similar strong concordance in a study where serology was compared to CD4 T cell responses using a CMV lysate as antigen preparation [25]. Whereas Sester et al. found that 100% of the seropositives gave *ex vivo* CD4 T cell responses to a CMV lysate, we found that 71% of the seropositives gave *ex vivo* CD4 T cell responses to a mixture of pp65 peptides. Partly explaining this difference is the fact that the pp65 protein, albeit being immunodominant, is only one of the more than 150 proteins, which have been shown to be immunogenic for CD4+ and/or CD8+ T cells [14]. Indeed, CMV lysates yield stronger CD4 T cell responses than mixture of pp65 peptides (unpublished observation). Sester *et al.* found that the few cases of false positives could be resolved by repeating the serological analysis whereby the seronegative assignment could be changed to seropositive suggesting that the T cell approach might actually be slightly more reliable that the serological approach. In contrast, our serological assignment remained unaltered when we repeated the serological analysis of the seronegative donors (data not shown). The major advantage of using the pp65 peptide mixture compared to a lysate is that it allows CD4 and CD8 T cell responses to be examined in parallel, something that Sester et al. could not do. Others have also successfully used pp65 derived peptide mixtures to examine CMV-specific CD8 T cell responses [26], [27].

The most surprising finding of our study is the very high fraction (≈75%) of seronegative donors that can mount a pp65-reactive CD4 T cells response after a brief *in vitro* stimulation culture. One explanation of this seeming anomaly might be that the observed CD4 T cell responses are not due to the immune system being bona fide CMV experienced, but rather due to immune cross-reactions to one or more of the closely related herpes virus. Partially addressing this question, we analyzed the CD4 T cell responses towards a lysate of HHV6 virus (Advanced Biotechnologies Inc.). In no case did the CD4 T cell responses of the in vitro pp65 stimulated seronegative T cells respond to the HHV6 lysate, although they all responded to a CMV lysate (data not shown). This does not rule out that these CD4 T cell responses are the result of cross-reactions against other related infectious agents and it remains a possible explanation for the observed CD4 T cell responses. In this context it should be noted that antigenic cross-reactivity has been observed between related herpes virus [28], [29], [30] and between related flavivirus [31], [32].

**Figure 2:**
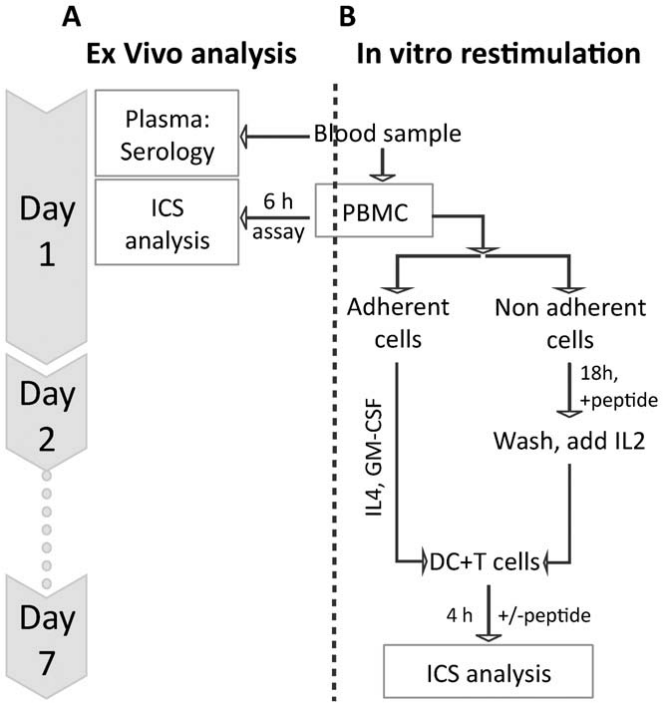
Flow diagram of the cell analysis. At day 1, some PBMC's were use directly for an *ex vivo* analysis of pp65 specific T cells. The remaining PBMC's were split in adherent and non-adherent cells. The non-adherent cells comprising the T cells were expanded on a pp65 peptide mixture. The peptide mixture was added overnight, washed away the next day, and the T cells were propagated on IL2 until day 7. At the same time, the adherent cells were cultured for 7 days in the presence of IL4 and GM-CSF to mature the into DC. At day 7, both the T cells and the DC's were harvested. The DC's were pulsed with the pp65 peptide mixture and added to the T cells (at the ratio 1∶10) during a 4 h ICS assay.

An alternative explanation for these findings could be that the in vitro peptide stimulation protocol has led to in vitro priming. We consider that unlikely since this would have required the presence of mature dendritic cells (DC's) [33], [34], [35], [36], [37], [38], [39]. PBMC's do not contain sufficient numbers of DC's to support in vitro priming. In fact, PBMC are noted for their “inability to support the sensitization of naïve antigen-specific lymphocytes under nearly all experimental conditions” [40]. Moreover, to avoid the risk of in vitro priming of naïve antigen-specific T cells, our in vitro expansion culture of non-adherent cells have on purpose been depleted of DC's. Rather, we separate DC and T cells allowing us to expand and mature a DC population in the absence of specific peptides, and to expand any specific memory T cell population in the presence of specific peptides. At the time of testing, the DC's are pulsed with specific peptides and only then mixed with the expanded T cells (the entire protocol is illustrated in Figure 2). Thus, our protocol is designed to minimize the risk that specific peptide, DC and T cells should all be present at the same time during the 7 day maturation and expansion period. To experimentally exclude that this in vitro stimulation procedure could lead to in vitro priming of pp65-specific CD4 T cell responses, five healthy donors were analyzed for responses against peptide mixtures derived from Yellow Fever virus. Since this virus is not endemic in our part of the world and the donors had never been vaccinated against Yellow Fever, we expected the immune system of our donors to be naïve with respect to Yellow Fever. None of the five naïve donors gave either CD4 or CD8 T cell responses against Yellow Fever derived peptides (data not shown). We conclude that the in vitro stimulation protocol used here is unable to perform in vitro priming.

Another alternative explanation might be that the classical serology criteria of CMV-experience could be skewed by the phenomenon of maternal antibody interference, in which specific maternal antibodies inhibits the development of specific antibody responses, but not that of T cell responses (see Text S1 for supplementary discussion of this phenomenon). This could affect the outcome of primary immunity against CMV-infections of the newborn and could potentially explain the very high frequency of CD4+ T cell responses in the absence of a humoral anti-CMV response. This scenario, however, raises other enigmas e.g. why would CMV reactivation and seroconversion in these individuals not have occurred later in life?

Our finding of frequent pp65-specific CD4 T cell reactivities in seronegative individuals may have some important prognostic implications for the selection of HCT donors and for the outcome of HCT. Seronegative donors, who mount an anti-pp65-specific, CD4+ T cell response (irrespective of how these have been generated), may be capable of mediating immune protection against CMV disease in the HCT recipient. CD4 T cells are known to be important for the control of CMV infection and reactivation. In healthy primary infected individuals, CD4 T cell responses precede antibody and CD8 T cell responses [41]. In HCT patients, CMV reactivation correlates with absence of CMV-specific CD4 T cells [3], [41], [42], [43], [44]. Transfer studies have shown that transfer of specific CD4 T cells can protect against CMV reactivation and disease [19], and specific CD8 T cells can clear an ongoing CMV infection, but not establish lasting immunity [21], [45]. Therefore, CMV-reactive CD4 T cells may contribute to a faster reestablishment of CMV immunity especially in HCT patients receiving a CMV seronegative donor graft. Thus, CMV-specific CD4 T cell responses (*ex vivo*, or after *in vitro* stimulation), rather than serology, might be a better risk indicator of CMV disease in allogeneic transplantations including HCT.

For the CMV-seronegative HCT recipient it may be preferable to use a CMV-seronegative donor to avoid primary CMV infection [4]. Intuitively, one would expect the use of a graft from a seropositive donor to be preferred to a CMV-seropositive recipient due to the potential for active transferred immunity. However, the data to support these hypotheses have been conflicting. CMV-seropositivity in the recipient is clearly associated with poorer outcome, but in some studies the survival curves for CMV-seropositive donors versus CMV-seronegative donors were super imposable for both CMV–positive and –negative recipients [4]. Our findings of a frequent occurrence of CMV reactive CD4 T cells in CMV seronegative individuals might explain these contradictory findings. CMV-seronegative donors, having highly cross-reactive and readily expandable CD4 T cells, might quickly be able to initiate competent CMV immunity. In our study we show that 75% of the seronegative individuals have CD4 T cells that after a brief *in vitro* stimulation can be expanded to high frequencies of up to 10%. Whether there is a correlation between donors with highly cross-reactive CD4 T cell responses and protection form CMV reactivation is currently under investigation.

From a diagnostic point of view, CMV-specific ex vivo T cell reactivity appears to be a sensitive and specific measure of CMV status, but in practical terms it does not offer any (at least not yet) advantages compared to serology. From a prognostic view, however, CMV-specific T cell reactivity, in particular after *in vitro* stimulation, may offer some important advantages. It may identify HCT donors capable of mounting a CMV-specific T cell response; donors, that would not have been identified by serology, and whose graft may afford T cell mediated CMV protection of the recipient. Thus, establishment of the cellular CMV status of donor and recipient may be an important criterion in the selection of the optimal HCT graft and other organ grafts. As the worldwide, unrelated donor registries continues to increase, the chances of identifying more than one donor for any given patient increases. Assessment of T cell mediated anti-CMV reactivity might improve donor selection and eventually the outcome of HCT.

## Materials and Methods

### Ethics Statement

The CMV study was approved at the National University Hospital of Copenhagen by “The Committees on Biomedical Research Ethics of the Capital Region” (Danish: “De Videnskabsetiske Komiteer for Region Hovedstaden”) (RH-3-CT5604). Informed written consent was obtained from 100 healthy blood donors.

The Yellow Fever study was approved at the University of Copenhagen by “The Committees on Biomedical Research Ethics of the Capital Region” (Danish: “De Videnskabsetiske Komiteer for Region Hovedstaden”) (H1-2009-095). Informed written consent was obtained from healthy blood donors.

### Peptides

Mixes of 134 peptides (15 amino acids long overlapping by 11 amino acids) spanning the entire CMV pp65 (UniProtKB/Swiss-Prot entry P06725, 561 amino acids long, 63 kDa) were obtained from JPT Peptide Technologies GmbH, Germany. For control purposes, mixes of 150 peptides (15 amino acids long overlapping by 11 amino acids) spanning parts of the proteome of an attenuated Yellow Fever vaccine (strain 17D-204) were obtained from Schafer-N, Copenhagen.

### Serological testing

Enzygnost® (Siemens Healthcare Diagnostics, Marburg, Germany, cat # OWGMG13) was used for qualitative detection of CMV-specific antibodies (total IgM and IgG) in donor plasma. The ELISA-kit was used according to the manufacturer's instructions. Briefly, microtiter plates that have been pre-coated with inactivated CMV antigen from human cell cultures are incubated with donor plasma. Bound IgG or IgM are simultaneously detected with peroxidase labeled anti-IgG and anti-IgM antibodies by addition of color substrate and reading by spectrometry. Results were interpreted as seropositive or seronegative as per manufacturer's instructions.

### Intracellular cytokine secretion analysis

Peripheral blood mononuclear cells (PBMC) were isolated from 30 ml freshly drawn blood by density gradient centrifugation (Ficoll-Paque, GE-Healthcare). The ex vivo and the in vitro stimulation assay strategies are illustrated on the left and right hand sides, respectively, of **Figure 2**.

For the *ex vivo* analysis, 2×10^6^ freshly isolated PBMC were stimulated with or without the pp65 peptide mix (2 µg/peptide/ml) for 6 h at 37°C. Brefeldin A (Sigma Aldrich) was present for the last 5 h of incubation.

Cells are incubated with 20 mM EDTA for 15 min, washed and permeablized in 200 µl 1% permeabilizing solution 2 (BD Biosciences) for 10 min. The cells were then washed and stained for 45 min at RT with 5 µL monoclonal anti-CD8 allophycocyanin (APC)-labelled, 10 µL anti-CD4 peridin chlorophyll protein (PerCP)-labeled, 10 µL anti-CD69 phycoerythrin (PE)–labeled, and 10 µL anti-IFNγ fluorescein isothiocyanate (FITC)-labeled and anti-TNFα FITC-labelled (BD PharMingen, San Diego, CA). Cells were washed with PBS, 1%BSA, 0.01%NaN_3_ and subsequently fixed in 1% formalin. The stained cells were analyzed on a FACS Calibur (BD Biosciences). 5–10*10^3^ CD4 and CD8 T cells were acquired.

The remaining PBMC's of the 30 ml blood sample were setup in a 7 days in vitro stimulation assay. To assure proper presentation of both MHC I and II restricted peptides in the ICS assay after 7 days of stimulation, APC's were propagated from the adherent cells while the non-adherent cells containing the T cells were used for in vitro stimulation (Figure 2). Thus the PBMC from the 30 ml blood sample were adhered in 24-well plates for 2 h at 37°C, and the adherent cells were cultured for 7 days in Xvivo15 with 5% AB serum, 100 ng/ml GM-CSF, and 100 ng/ml IL4. The non-adherent cells were harvested and incubated with 2 µg/ml/peptide pp65 peptide mix in Xvivo15 (Lonza) supplemented with 5% autologous serum. To obtain a specific stimulation with less background expansion the cells were harvested after 18–20 h incubation washe and plated in new wells with 50 U/ml IL2. Fresh media and IL2 were added at day 4, and 6. The DC and T cell cultures were harvested at day 7. The DC's were pulsed with or without pp65 peptide mix (2 µg/peptide/ml) for 1 h and then added to the T cells, at a 1∶10 ratio of DC:T cells. The cells were incubated for 4 h at 37°C. Brefeldin A (SigmaAldrich) was present for the last 3 h of incubation. We find that extending the incubation for more than 4 h induces proliferation when analyzing frequencies of specific T cells post expansion. The cells were then stained and analyzed by flow cytometry as described above.

The frequency of cytokine producing T cells were determined. The results are given as frequency of specific cytokine producing T cells subtracted the background frequency of cytokine producing T cells in the “no peptide” sample - 0.09% for the ex vivo analysis and 0.3% for the in vitro stimulated samples. Responses 2 times above background (thus one time above background for the subtracted values) were considered positive. All FACS plots were subsequent evaluated for whether the negative donors by this definition were truly negative and the positive donors truly positive. The intensity of the cytokine staining were lower for all donors with a frequency below 2 times the background level than for donors above this threshold.

For control purposes, the in vitro stimulation analysis was repeated on PBMC's obtained from 5 healthy naïve donors, however, substituting the CMV pp65 peptide mixture with similar peptides mixtures (same sampling strategy (15 amino acid peptide length, 11 amino acid overlap), similar sampling size (150 peptides/pool) and same peptide concentrations (2 µg/ml/peptide)) derived from the proteome of the Yellow Fever 17D-204 vaccine.

## Supporting Information

Text S1 **Supplementary discussion and references.**
(DOCX)
