# Supplementary material for: Humoral and Cellular CMV Responses in Healthy Donors; Identification of a Frequent Population of CMV-Specific, CD4+ T Cells in Seronegative Donors
Source: PLoS One. 2012 Feb 7;7(2):e31420. doi: 10.1371/journal.pone.0031420 (PMC3274531; doi:10.1371/journal.pone.0031420)
Supplement: Text S1 — Supplementary discussion and references. (DOCX) [file pone.0031420.s001.docx]

## S1. Supplementary discussion

Indeed, maternally transferred anti-CMV antibodies have been suggested to explain why CMV seroprevalence among infants less than 6 months of age are higher than that of older children (this exceptional trend has been found in four different studies [1,2,3,4] and has been interpreted as reflecting “the transient presence of maternally acquired passive antibodies among infants”[5]). In fact, it has been reported that even minute amounts of specific maternal antibodies against bovine herpes virus 1 (BHV) in calves blocks the development of a humoral response upon vaccination against BHV [6]. It has also been reported that specific maternal antibodies against bovine viral diarrhoea virus (BVDV) in calves block the development of a specific antibody response, but not the development of protective CD4+ and CD8+ T cell responses, nor the development of memory B cell responses [7]. Maternal antibody mediated inhibition has even been observed in primate systems. Thus, when experimental measles virus vaccination of cynomolgous macaques was performed in the presence of passively transferred macaque serum against measles virus the induction of specific IgG and IgM antibodies was completely blocked, whereas the induction of protective immunity - probably mediated by specific T cells - was not blocked [8].

## References for Supplementary discussion

1. Almeida LN, Azevedo RS, Amaku M, Massad E (2001) Cytomegalovirus seroepidemiology in an urban community of Sao Paulo, Brazil. Rev Saude Publica 35: 124-129.

2. (1970) Cytomegalovirus infection in the north west of England. A report on a two-year study. Arch Dis Child 45: 513-522.

3. Deibel R, Smith R, Clarke LM, Decher W, Jacobs J (1974) Cytomegalovirus infections in New York State. N Y State J Med 74: 785-791.

4. Embil JA, Haldane EV, MacKenzie RA, van Rooyen CE (1969) Prevalence of cytomegalovirus infection in a normal urban population in Nova Scotia. Can Med Assoc J 101: 78-81.

5. Cannon MJ, Schmid DS, Hyde TB Review of cytomegalovirus seroprevalence and demographic characteristics associated with infection. Rev Med Virol 20: 202-213.

6. Brar JS, Johnson DW, Muscoplat CC, Shope RE, Jr., Meiske JC (1978) Maternal immunity to infectious bovine rhinotracheitis and bovine viral diarrhea viruses: duration and effect on vaccination in young calves. Am J Vet Res 39: 241-244.

7. Endsley JJ, Roth JA, Ridpath J, Neill J (2003) Maternal antibody blocks humoral but not T cell responses to BVDV. Biologicals 31: 123-125.

8. Osterhaus A, van Amerongen G, van Binnendijk R (1998) Vaccine strategies to overcome maternal antibody mediated inhibition of measles vaccine. Vaccine 16: 1479-1481.
